# Comparison of cognitive performance between patients with Parkinson’s disease and dystonia using an intraoperative recognition memory test

**DOI:** 10.1038/s41598-021-99317-6

**Published:** 2021-10-20

**Authors:** Lin Shi, Tianshuo Yuan, Shiying Fan, Yu Diao, Guofan Qin, Defeng Liu, Guanyu Zhu, Kai Qin, Huanguang Liu, Hua Zhang, Anchao Yang, Fangang Meng, Jianguo Zhang

**Affiliations:** 1 Department of Neurosurgery, Beijing Tiantan Hospital, Capital Medical University, Beijing, China; 2 Department of Functional Neurosurgery, Beijing Neurosurgical Institute, Capital Medical University, Beijing, China; 3 Alpha Omega Engineering Ltd., Nazareth, Israel

**Keywords:** Learning and memory, Parkinson's disease

## Abstract

Neuroscientific studies on the function of the basal ganglia often examine the behavioral performance of patients with movement disorders, such as Parkinson’s disease (PD) and dystonia (DT), while simultaneously examining the underlying electrophysiological activity during deep brain stimulation surgery. Nevertheless, to date, there have been no studies comparing the cognitive performance of PD and DT patients during surgery. In this study, we assessed the memory function of PD and DT patients with the Montreal Cognitive Assessment (MoCA) and the Mini-Mental State Examination (MMSE). We also tested their cognitive performance during the surgery using a continuous recognition memory test. The results of the MoCA and MMSE failed to reveal significant differences between the PD and DT patients. Additionally, no significant difference was detected by the intraoperative memory test between the PD and DT patients. The intraoperative memory test scores were highly correlated with the MMSE scores and MoCA scores. Our data suggest that DT patients perform similarly to PD patients in cognitive tests during surgery, and intraoperative memory tests can be used as a quick memory assessment tool during surgery.

## Introduction

Parkinson’s disease (PD) is a neurodegenerative disorder characterized by resting tremor, bradykinesia and muscular rigidity^1^. Pathologically, motor dysfunctions associated with PD are thought to result primarily from the loss of dopaminergic neurons in the substantia nigra^2^, while neurons in other parts of the brainstem and basal ganglia are also found to be in the process of degeneration, which contributes to a complex of nonmotor symptoms in patients with PD, including dermatological, automatic, neurobehavioral, and sensory disorders^3^. Many studies have reported cognitive dysfunction in those patients, such as symptoms related to executive functions, language, memory and psychological features which severely affect patient quality of life^3–6^. Among these symptoms, memory deficits in PD seem to be the most complicated because many studies have reported contradictory results. It is not difficult to understand that PD patients may experience nondeclarative memory deficits due to degeneration in the basal ganglia, which is believed to play a role in the nondeclarative memory system^7–9^. Recent evidence from PD studies tends to indicate that PD patients also suffer from deficits in declarative memory that are traditionally thought to be mediated by the mesial temporal lobe system^4,6,7,10^. However, to date, the exact type of memory deficit and its related mechanisms have remained unclear^4,5^.

On the other hand, primary dystonia (DT), a kind of movement disorder featuring repetitive movements and stereotyped abnormal postures produced by involuntary contraction of muscles, is also considered to be related to dysfunction in the basal ganglia^11^. Studies have shown that DT patients also experience cognitive dysfunction, including deficits in memory, processing speed, and decision making^12–15^. Nevertheless, research on memory deficits in DT patients is still very limited.

Deep brain stimulation (DBS) surgery is a well-accepted therapeutic modality for PD and DT patients. It is also a good opportunity to examine patients’ behavioral performance while simultaneously studying the electrophysiological activity of the basal ganglia during microelectrode recording^16,17^. A few studies have been performed to investigate memory functions in PD and DT patients undergoing DBS surgery using this method^16–18^. PD and DT are related but differ in clinical presentation, pathological genesis, and electrophysiological features. It is therefore of great interest for both theoretical and clinical purposes to assess and compare memory behavior of PD and DT patients during DBS surgery, which will contribute to the investigation of the functions of the basal ganglia in the memory circuit. However, to date, there have been no studies or reports comparing memory performance of PD and DT patients. In this preliminary study, we compared the cognitive performance in PD and DT patients during DBS surgery, and correlated the intraoperative cognitive performance with preoperative cognitive assessments.

## Methods

### Patients and ethics statement

The data presented herein were obtained from patients undergoing bilateral DBS surgery for the treatment of PD or DT at the clinic of the Movement Disorder Center in Beijing Tiantan Hospital between 2019 and 2021. All patients met the existing selection criteria for DBS therapy in China^19^, including those with suspicion of mild dementia (either MMSE = 18–23 or MoCA = 11–17, but not both). These patients were not excluded from DBS therapy based on the following considerations: (1) dementia is a comprehensive diagnosis, and the fact that either the MMSE or MoCA score is within the range of mild dementia is not an absolute contraindication for DBS; (2) these patients can behave quite normally in daily life and express strong desire to have DBS surgery even if being informed of the potential risks of long-term cognitive decline; (3) there is not sufficient evidence that STN-DBS or GPi-DBS will lead to cognitive impairment that is independent of advanced age^3,20^. In this study, patients with definitive dementia or severe anxiety or depression, age over 75, Hoehn-Yahr scale over 4.0, or other systemic diseases were excluded.

A total of 51 patients volunteered to participate in the study, including 22 PD patients (11 males and 11 females) and 18 DT patients (8 males and 10 females). The average age at surgery was 57.9 ± 8.4 years for the PD patients and 54.4 ± 9.5 years for the DT patients (P = 0.203). The average duration of disease was 8.4 ± 3.9 years in the PD patients and 7.6 ± 4.1 years in the DT patients (P = 0.157). The dominant symptoms included tremor, rigidity and bradykinesia in the PD patients and torsion, torticollis, and Meige’s syndrome in the DT patients. The educational background of the patients was balanced. Details of the demographic and clinical data of the patients are listed in Tables 1 and S1 in supplementary material. All patients were informed of the aims and procedures of the present study and signed informed consent forms. The study was performed in accordance with the Declaration of Helsinki, and all protocols were approved by the ethics committees of Beijing Tiantan Hospital (reference code: KY2019-097-02).

**Table 1:** Summary of demographic and clinical characteristics of the PD and DT patients in this study.

	Number of patients/sex	Age (yrs)	Duration (yrs)	LEDD (mg)	mUPDRS (off/on) or UDRS	BFMDRS	H-Y scale	HAMA	HAMD	SF-36	MMSE	MoCA	Preop test score (%)	Intraop Test score (%)	Target of DBS
PD	22 (11 m/11 f)	57.9 ± 8.4	8.4 ± 3.9	747.9 ± 285.8	34.4 ± 10.6 /17.5 ± 6.9	–	2.6 ± 0.7	11.9 ± 4.2	12.1 ± 5.6	72.2 ± 9.4	26.7 ± 2.5	24.2 ± 4.8	75.4 ± 12.6	73.8 ± 15.1	14 STN, 8 GPi
DT	18 (8 m/10 f)	54.4 ± 9.5	7.6 ± 4.1	–	16.8 ± 7.3	11.2. ± 3.8	–	10.9 ± 5.4	11.2 ± 5.6	68.3 ± 11.8	27.6 ± 1.9	22.3 ± 4.8	76.6 ± 11.7	80.3 ± 13.6	10 STN, 8 GPi

### Preoperative assessments

The diagnosis of PD and DT in these patients was confirmed by a neurologist. All patients underwent neurological and neuropsychological assessments, including the Hoehn-Yahr scale and the Unified Parkinson’s Disease Rating Scale (UPDRS, on/off medication) for PD patients and the Unified Dystonia Rating Scale (UDRS) and Burke-Fahn-Marsden Dystonia Rating Scale (BFMDRS) for DT patients. The motor scores of the UPDRS (mUPDRS) and UDRS (mUDRS) were used for comparison in this study. In both patient groups, the short-form health survey 36-item scale (SF-36) was used to assess overall quality of life. The Hamilton rating scale for depression (HAMD) and Hamilton rating scale for anxiety (HAMA) were used to evaluate the patients’ psychological conditions. The cognition of the PD and DT patients was examined using the Montreal Cognitive Assessment (MoCA) and Mini-Mental State Examination (MMSE). Detailed scores from the preoperative assessments are listed in Tables 1 and S1 in Supplementary Material.

*BFMDRS* Burke-Fahn-Marsden dystonia rating scale, *DT* dystonia, *HAMA* Hamilton rating scale for anxiety, *HAMD* Hamilton rating scale for depression, *H-Y scale* Hoehn-Yahr scale, *LEDD* levodopa equivalent daily dose, *MMSE* Mini-Mental State Examination, *MoCA* Montreal Cognitive Assessment, *PD* Parkinson’s disease, *S* sedatives, *SF-36* short-form health survey 36-item scale, *mUPDRS* motor score of the Unified Parkinson’s Disease Rating Scale, *mUDRS* motor score of the Unified Dystonia Rating Scale.

### Surgical procedures

A preoperative 3.0 T brain MRI scan was performed at admission according to a standard protocol^21^. The planning of trajectories for DBS surgery was conducted using the direct targeting method as described elsewhere^21^. Patients were off their medication for at least 12 h prior to surgery. All patients received bilateral DBS surgery on the operation day. The procedures of the surgery were described in detail in previous reports^22^. Briefly, local anesthesia was administered using lidocaine plus ropivacaine, and no other anesthetics were applied so that the patient remained fully awake during the surgery while pain was minimized. No patient reported any severe discomfort. After disinfecting the scalp, an incision and a burr hole were made along the predetermined trajectories. A microelectrode (Alpha Omega, Israel) was driven into the brain to the target area (STN or GPi, see Tables 1 and Table S1 in supplementary material for detailed information) by a microdrive. Electrophysiological signals were recorded to reveal the entrance to and exit from the STN and GPi, which aid the implantation of DBS leads. When a standard STN or GPi signal was identified, a continuous recognition memory test was performed to test the patient’s cognition, as described below. Then, stimulation effectiveness and side effects were tested by neurosurgeons using a temporary stimulator. If the effects of temporary stimulation were satisfactory in PD patients, a permanent stimulator was then implanted, while in DT patients, stimulation effectiveness was tested in the ward for several days to decide whether to implant the permanent stimulator. Other procedures of the surgery were similar to other descriptions of standard DBS surgeries^23,24^.

### Continuous recognition memory test

**Figure 1:**
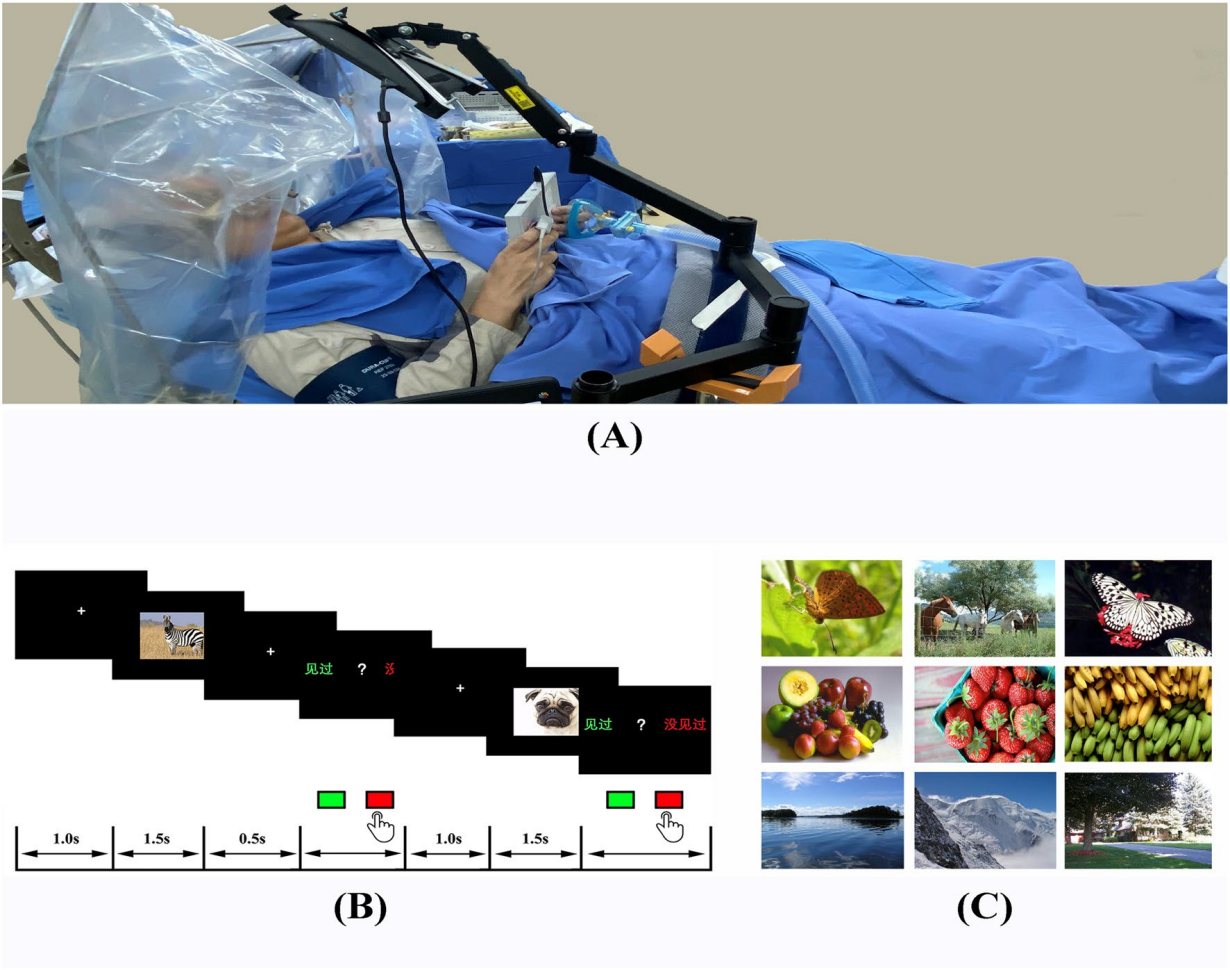
The memory test used in the study. (**A**) Devices, patient position and settings during the memory test. (**B**) Upper panel, screens presented to the patients during an example trial. Lower panel, the lengths of time for which each screen was shown. (**C**) Sample images used in the test.

The test used in this study was a continuous recognition memory test (Fig. 1). The test consisted of a series of 108 images, 54 of which were unique. All images were randomly chosen from an online image database^25^. The images were shown on an LCD screen placed in front of the patient. Each trial started with a fixation cross shown for 0.5–1.0 s (randomized). Afterwards, an image was shown for 1.5 s. Each image belonged to one of three categories (evenly distributed among animals, landscapes, and fruits). After image offset, a second fixation cross was shown for 0.5 s, followed by a question that remained on the screen until the patient made a response (no timeout). The patient was instructed by the question to categorize each image as novel or familiar with a response pad (RB-844, Cedrus Inc, California, USA.) by pressing the red or green button on the pad. The test was similar to that described previously^17^, except that we used a Chinese version of the test. The test was implemented in MATLAB using the PsychToolbox package^26^. On the night before surgery, the patient was trained with a short version of the test. On the operation day, the patients were tested in the ward for their memory baseline. During DBS surgery, when the microelectrode was in place, the test was performed to test the patients’ memory in the operating room (OR). For each subject, the test took approximately 10 min. All patients cooperated very well during the entire test. No patients reported an inability to finish or any discomfort during the test.

### Statistical methodology

We compared the MoCA scores, MMSE scores, and intraoperative memory test scores to evaluate cognitive behavior of the PD and the DT patients. We also compared the intraoperative memory test scores between the PD and DT patients in subgroups based on cognitive impairment defined by the MoCA scale^6,27,28^, including a subgroup with high MoCA scores (26–30, indicating normal cognition), a subgroup with medium MoCA scores (18–25, indicating mild cognitive impairment), and a subgroup with low MoCA scores (11–17, indicating mild dementia). This grouping criterion is in line with the cognitive impairment levels defined by the MoCA scale^29^, which is believed to be superior to the MMSE in detecting cognitive changes^30–32^. Correlation analyses were performed to evaluate the relationships between the intraoperative memory test scores and the preoperative cognitive assessment scores. Descriptive statistics are reported as the means and standard deviations. Student’s t test was used to compare variables between the PD and DT patients. Statistical analyses were performed using MATLAB 2018b (Mathworks, USA). The significance level was set at an alpha of *P* < 0.05.

### Consent for publication

All authors signed the consent for publication.

## Results

### Preoperative MMSE and MoCA scores

**Figure 2:**
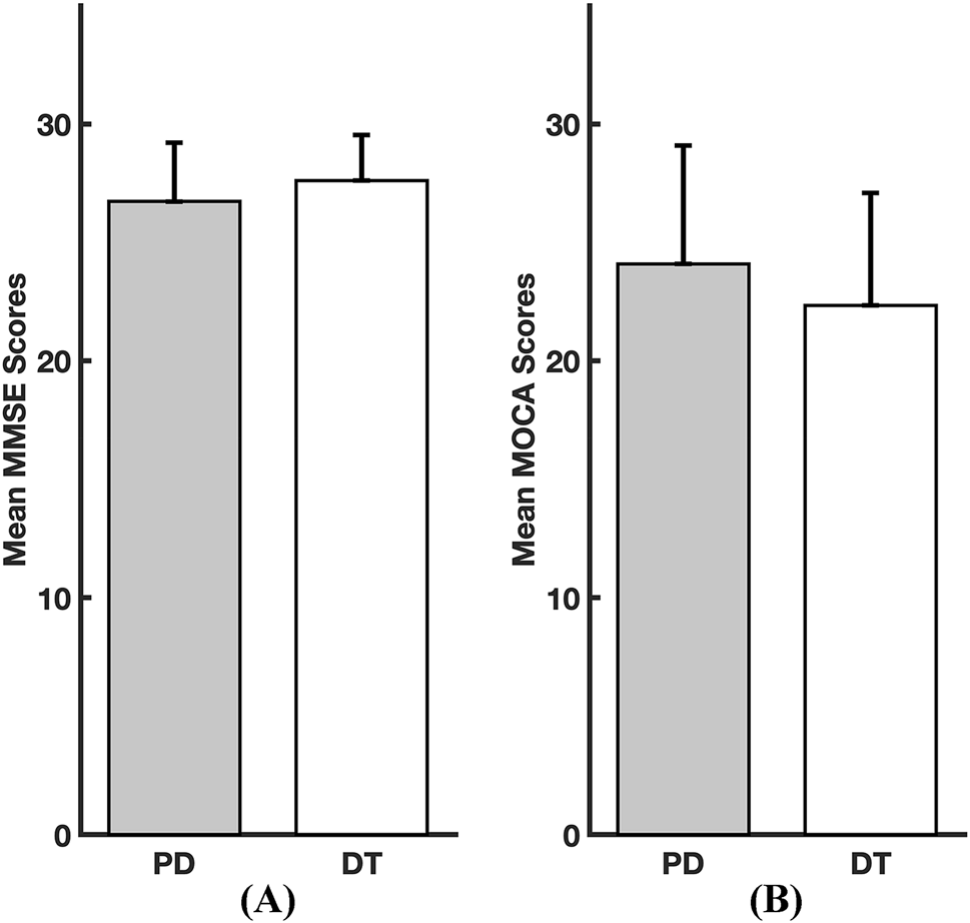
Comparisons of the MMSE scores and the MoCA scores between the PD and DT patients. (**A**) Comparison of mean MMSE scores between the PD patients and the DT patients. (**B**) Comparison of the mean MoCA scores between the PD patients and the DT patients. *MMSE* Mini-Mental State Examination, *MoCA* Montreal Cognitive Assessment, *PD* Parkinson’s disease; DT, dystonia.

In this study, we evaluated the cognitive function of 22 PD patients and 18 DT patients before and during DBS surgery. The preoperative MMSE scores were 26.7 ± 2.5 in the PD patients and 27.6 ± 1.9 in the DT patients. The preoperative MoCA scores were 24.2 ± 4.8 in the PD patients and 22.3 ± 4.8 in the DT patients. Of the PD patients, 7 were in the high MoCA subgroup (31.8%), 11 were in the medium MoCA subgroup (50.0%) and 4 were in the low MoCA subgroup (18.2%); of the DT patients, 3 were in the high MoCA subgroup (16.7%), 12 were in the medium MoCA subgroup (66.7%), and 3 were in the low MoCA subgroup (16.7%). No significant differences were found between the PD and DT patients in the preoperative MMSE scores (*P* = 0.222, Fig. 2A) or MoCA scores (*P* = 0.264, Fig. 2B), indicating that the overall preoperative cognitive distribution of the PT and DT patients was balanced.

### Preoperative and intraoperative memory test scores

**Figure 3:**
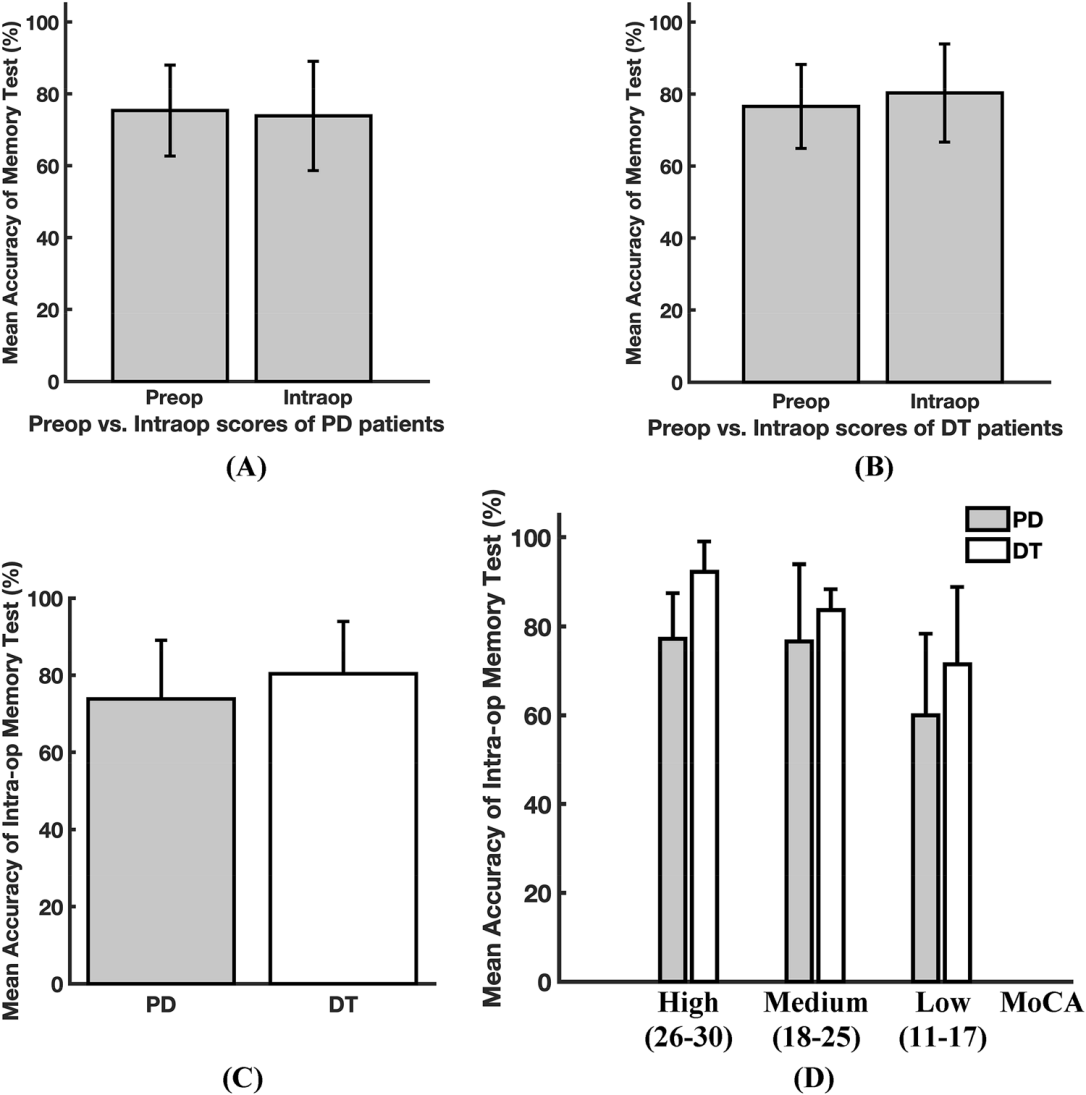
Comparison of the intraoperative memory test scores between the PD and DT patients. (**A**) Comparison of the preoperative and intraoperative memory test scores in the PD patients. (**B**) Comparison of the preoperative and intraoperative memory test scores in the DT patients. (**C**) Comparison of the intraoperative memory test scores between the PD and DT patients. (**D**) Comparison of the intraoperative memory test scores between the PD and DT patients in cognitive impairment subgroups as defined by the MoCA scores^27,28^. *PD* Parkinson’s disease, *DT* dystonia, *MoCA* Montreal Cognitive Assessment.

We tested the memory of the PD and DT patients before DBS surgery using the aforementioned memory test. The preoperative memory test scores were 75.4 ± 12.6% for the PD patients and 76.6 ± 11.7% for the DT patients (Fig. 3A,B). During DBS surgery, when the microelectrode was placed in the STN/GPi, the patients were retested with the test. The intraoperative memory test scores were 73.8 ± 15.1% for the PD patients and 80.3 ± 13.6% for the DT patients (Fig. 3A,B). No significant differences were detected between the PD and DT patients (all *P*s > 0.05, Fig. 3C), either in the preoperative test scores or in the intraoperative test scores, and there were no significant differences between the preoperative and intraoperative test scores (all *P*s > 0.05, Fig. 3A,B), indicating that the patients’ performance was consistent before and during the surgery. Moreover, the intraoperative test scores were compared between the PD and DT patients with high (26–30), medium (18–25), and low (11–17) MoCA scores. Similarly, there were no significant differences detected in the intraoperative memory test scores between the PD and DT patients (all *P*s > 0.05, Fig. 3D).

### Correlation of the intraoperative memory test scores with the MMSE and MoCA scores

**Figure 4:**
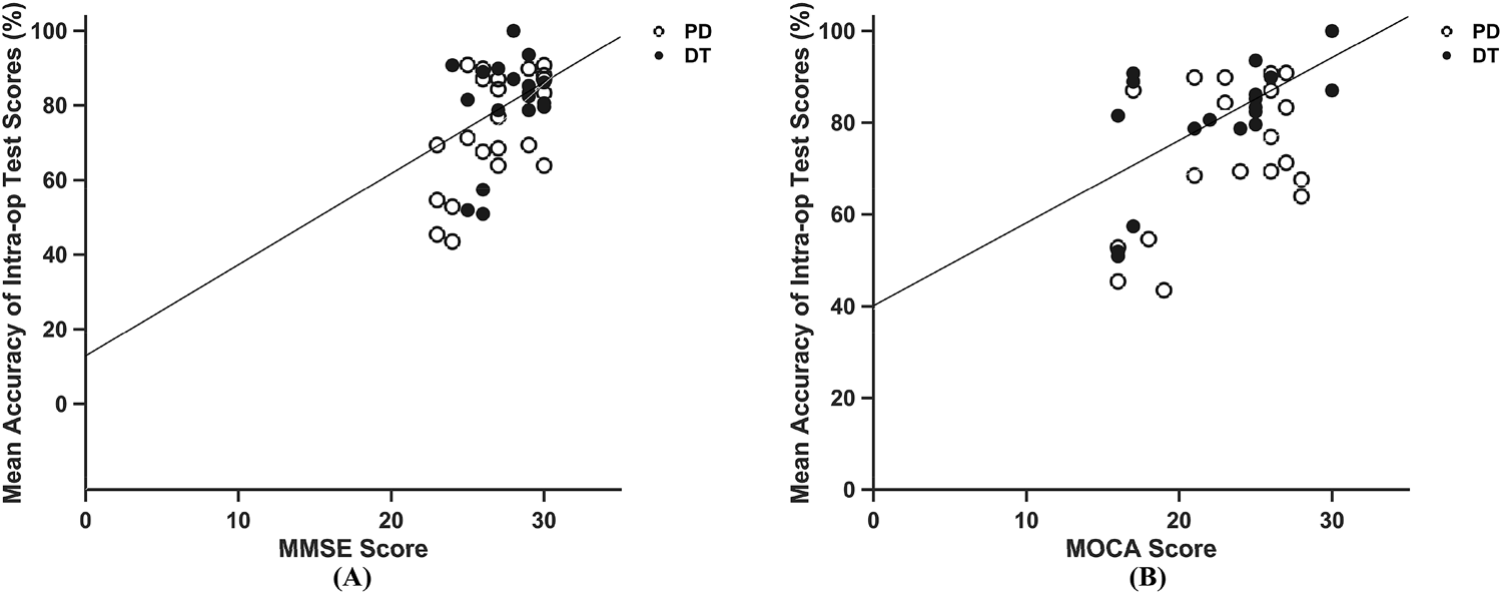
Correlation of the intraoperative memory test scores with the MMSE and MoCA scores. (**A**) Correlation of the intraoperative memory test scores with the MoCA scores. (**B**) Correlation of the intraoperative memory test scores with the MMSE scores. *MoCA* Montreal Cognitive Assessment, *MMSE* Mini-Mental State Examination, *PD* Parkinson’s disease, *DT* dystonia.

To further evaluate the function of the intraoperative memory test in cognitive assessment, we correlated the intraoperative test scores with the preoperative cognitive assessment results, i.e., the MMSE scores and MoCA scores. We found that the intraoperative memory test scores were highly correlated with the MMSE scores (*R* = 0.521, *P* = 0.0006, Fig. 4A) and MoCA scores (*R* = 0.472, *P* = 0.002, Fig. 4B). These results suggested that the scores of the intraoperative memory test may, to some extent, reflect the results of the preoperative cognitive assessments, including the MMSE and the MoCA scores.

## Discussion

In the present study, we assessed the cognitive deficits in 22 PD and 18 DT patients using a recognition memory test during DBS surgery, and correlated the results with preoperative assessments. To the best of our knowledge, no other research has compared the cognitive behavior of PD and DT patients in the OR using a modern trial-based quantitative method. The test used in our study was a continuous recognition memory test, and thus, the accuracy of the test can to some extent roughly reflect episodic memory function, which are memories of day-to-day events of life^6^. Our results showed that in both PD and DT patients, the percentage of patients whose MoCA scores fell in the range of mild dementia was approximately 16–18%, which is higher than the estimated proportion of dementia in the general population (5–8%)^33,34^. This finding is in accordance with previous studies that have reported recognition memory impairment in PD patients^6,35^ and shows that cognitive impairment may exist in DT patients.

The memory test failed to detect significant differences between the PD and DT patients, either by comparison of test accuracy between the entire PD and DT groups or by comparison of test accuracy in the subgroups defined by the MoCA scale^28,29^ (Fig. 3). That is, our data showed that the memory test performance of DT patients and PD patients who fell in the same MoCA category was equal during the operation. This is important because PD and DT are physiologically and pathologically different. To reveal the underlying mechanisms, comparing neuronal activity in the basal ganglia between PD and DT patients in experimental brain research conditions may be a good attempt, and our data support this idea because the memory behavior between the PD and DT patients was comparable.

The MoCA and MMSE scales are widely used screening assessments for detecting cognitive impairments in multiple neurodegenerative diseases^28^. Our data show that the intraoperative test accuracy correlated well with the preoperative MoCA and MMSE scores, suggesting that the intraoperative memory test can, to some extent, roughly reflect the cognitive conditions. In other words, it can be used as a quick cognition assessment tool during surgery. It is not clear whether DBS influences cognition, and previous studies have reported contradictory results^3,20,36,37^. With such a quick assessment method, it is convenient to test the patients under local anesthesia during DBS surgery and determine whether there are dramatic changes in the patient’s cognitive performance. This is similar to the effect of routine intraoperative test stimulation on motor function. If the test accuracy clearly decreases from preoperative accuracy, it is reasonable to consider the necessity of adjusting the coordinates of the target.

A minor finding from our study was that the intraoperative test accuracy was equal to the preoperative test accuracy. This finding confirms that patient performance may not be greatly influenced in the OR during awake surgery by the setting of the OR and by the surgical procedures.

Our study has some limitations. In the test, only basic recognition memory function was assessed, and therefore, the results are not sufficient to detect changes in other cognitive domains. The test can only be taken as a rough reflection of cognition. Another limitation of our study is that the comparison of cognitive performance during DBS surgery between Parkinson’s disease and dystonia patients is slightly doubtful. However, we believe it is reasonable to do so because the overall clinical characteristics of these patients were balanced, including age, disease duration, education, preoperative cognitive assessments, surgical procedures, settings, etc. Moreover, the population in each group was not very large, and thus the patients in the STN and GPi groups were not separately analyzed. Therefore, a better-designed standardized experiment needs to be performed to overcome these issues.

In conclusion, our data suggest that the performance of DT patients is generally comparable to that of PD patients during DBS surgery, and the results of the intraoperative memory test correlate with the preoperative MoCA and MMSE scores.

## Supplementary Information

Supplementary Information.

## Data Availability

The data that support the findings of this study are available from the corresponding authors upon request.

## Code availability

The custom codes that support the findings of this study are available from the corresponding authors upon request.
