# Author Correction: Comparison of cognitive performance between patients with Parkinson’s disease and dystonia using an intraoperative recognition memory test

**DOI:** 10.1038/s41598-021-04332-2

**Published:** 2021-12-27

**Authors:** Lin Shi, Tianshuo Yuan, Shiying Fan, Yu Diao, Guofan Qin, Defeng Liu, Guanyu Zhu, Kai Qin, Huanguang Liu, Hua Zhang, Anchao Yang, Fangang Meng, Jianguo Zhang

**Affiliations:** 1 Department of Neurosurgery, Beijing Tiantan Hospital, Capital Medical University, Beijing, China; 2 Department of Functional Neurosurgery, Beijing Neurosurgical Institute, Capital Medical University, Beijing, China; 3 Alpha Omega Engineering Ltd, Nazareth, Israel

Correction to: *Scientific Reports* 10.1038/s41598-021-99317-6, published online 20 October 2021

The original version of this Article contained an error in the author list. Jie Zheng was incorrectly listed as an author of the original Article, and has subsequently been removed.

The Author Contributions section now reads:

L.S. and J.Zha. conceived and designed the study. L.S., T.Y., S.F., D.L., K.Q., H.L. and H.Z. collected the data. L.S., T.Y., S.F., D.L., Y.D., H.L. and H.Z. analyzed the data. Y.D., S.F., G.Q., G.Z. and A.Y. created the figures. L.S., T.Y., A.Y. and F.M. wrote the draft of the manuscript. L.S., S.F., D.L., K.Q. and Y.D. revised the manuscript. All authors approved the submission of the manuscript and the revised manuscript.

The original Article has been corrected.

