# Supplementary material for: Comparison of cognitive performance between patients with Parkinson’s disease and dystonia using an intraoperative recognition memory test
Source: Sci Rep. 2021 Oct 20;11:20724. doi: 10.1038/s41598-021-99317-6 (PMC8528828; doi:10.1038/s41598-021-99317-6)
Supplement: Supplementary file 1 — Supplementary Information. [file 41598_2021_99317_MOESM1_ESM.docx]

**Supplementary Material**

**Table S1. Demographic and clinical characteristics of the patients in this study.**

| Case No. | Sex/Age (yrs) | Duration (yrs) | DT Type | Cause | Affected Regions | Motor Symptoms | Previous Treatment | DBS Target | LEDD (mg) | mUPDRS (off/on) | BFMDRS/mUDRS | H-Y Scale | HAMA | HAMD | SF-36 | MMSE | MoCA | Preop Test Score | Intraop Test Score |
| --- | --- | --- | --- | --- | --- | --- | --- | --- | --- | --- | --- | --- | --- | --- | --- | --- | --- | --- | --- |
| PD01 | M/52 | 12 | - | I | limbs | tremor, rigidity | APD | STN | 812 | 42/20 | - | 2.0 | 11 | 7 | 65 | 25 | 27 | 0.639 | 0.713 |
| PD02 | M/63 | 8 | - | I | limbs | tremor, rigidity | APD, AD | STN | 868 | 26/10 | - | 2.0 | 21 | 19 | 62 | 23 | 24 | 0.843 | 0.694 |
| PD03 | M/69 | 13 | - | I | limbs, trunk | tremor, rigidity, FOG | APD | STN | 1000 | 41/24 | - | 3.0 | 11 | 16 | 74 | 27 | 28 | 0.639 | 0.639 |
| PD04 | F/65 | 6 | - | I | limbs | tremor, rigidity | APD | STN | 781 | 47/27 | - | 2.0 | 12 | 15 | 72 | 27 | 23 | 0.713 | 0.843 |
| PD05 | M/64 | 5 | - | I | limbs | tremor | APD | GPi | 300 | 21/10 | - | 2.0 | 7 | 4 | 86 | 30 | 27 | 0.908 | 0.880 |
| PD06 | F/54 | 15 | - | I | limbs | tremor, rigidity | APD, S | STN | 900 | 48/18 | - | 2.5 | 11 | 12 | 65 | 26 | 23 | 0.769 | 0.898 |
| PD07 | M/66 | 16 | - | I | limbs | tremor | APD | STN | 750 | 43/24 | - | 2.0 | 10 | 11 | 72 | 29 | 21 | 0.685 | 0.898 |
| PD08 | F/46 | 8 | - | I | limbs | tremor, rigidity, BK | APD, N | GPi | 950 | 50/18 | - | 2.5 | 8 | 2 | 84 | 27 | 26 | 0.907 | 0.870 |
| PD09 | F/52 | 4 | - | I | limbs, trunk, voice | tremor, rigidity, FOG, BK | APD, AD | STN | 800 | 48/20 | - | 4.0 | 18 | 18 | 81 | 30 | 26 | 0.898 | 0.676 |
| PD10 | F/50 | 7 | - | I | limbs, trunk | tremor, rigidity, FOG | APD | STN | 1075 | 47/27 | - | 3.0 | 13 | 19 | 80 | 26 | 28 | 0.769 | 0.454 |
| PD11 | M/66 | 6 | - | I | limbs | tremor, rigidity, BK | APD | GPi | 400 | 31/18 | - | 2.5 | 7 | 5 | 65 | 23 | 19 | 0.769 | 0.639 |
| PD12 | M/51 | 8 | - | I | limbs, trunk | tremor, rigidity, BK | APD, N | STN | - | 38/25 | - | 3.0 | 11 | 14 | 52 | 30 | 28 | 0.528 | 0.685 |
| PD13 | F/54 | 5 | - | I | limbs | tremor, rigidity | APD | GPi | 800 | 14/8 | - | 2.5 | 18 | 10 | 54 | 27 | 21 | 0.908 | 0.870 |
| PD14 | F/62 | 10 | - | I | limbs | tremor, rigidity | APD, N | STN | 600 | 31/15 | - | 2.0 | 8 | 15 | 82 | 26 | 17 | 0.713 | 0.546 |
| PD15 | F/64 | 11 | - | I | limbs | rigidity, BK | APD | STN | 1410 | 28/14 | - | 4.0 | 10 | 14 | 70 | 23 | 18 | 0.898 | 0.694 |
| PD16 | M/42 | 10 | - | I | limbs | tremor, rigidity | APD | GPi | 300 | 30/14 | - | 2.5 | 9 | 5 | 65 | 29 | 26 | 0.639 | 0.833 |
| PD17 | M/55 | 6 | - | I | limbs | tremor, rigidity | APD | GPi | 750 | 26/10 | - | 2.0 | 8 | 14 | 72 | 30 | 27 | 0.528 | 0.435 |
| PD18 | F/63 | 3 | - | I | limbs | tremor | APD, AD | GPi | 300 | 28/10 | - | 2.0 | 18 | 23 | 84 | 24 | 19 | 0.898 | 0.908 |
| PD19 | F/61 | 5 | - | I | limbs | tremor | APD | STN | 734 | 18/8 | - | 2.0 | 17 | 13 | 78 | 25 | 27 | 0.713 | 0.528 |
| PD20 | M/66 | 15 | - | I | limbs, trunk | tremor, rigidity, BK | APD | STN | 600 | 31/19 | - | 3.0 | 7 | 7 | 80 | 24 | 16 | 0.639 | 0.769 |
| PD21 | F/54 | 7 | - | I | limbs, trunk, voice | rigidity, FOG， BK | APD | GPi | 1075 | 41/32 | - | 4.0 | 14 | 17 | 72 | 27 | 26 | 0.676 | 0.907 |
| PD22 | M/54 | 4 | - | I | limbs | tremor, rigidity | APD | STN | 500 | 28/14 | - | 2.0 | 12 | 7 | 74 | 30 | 26 | 0.907 | 0.852 |
| DT01 | F/72 | 8 | writing spasm | I | limbs | torsion | BT, N | STN | - | - | 11/14 | - | 12 | 6 | 76 | 26 | 16 | 0.574 | 0.509 |
| DT02 | M/47 | 4 | torticollis | I | neck | torsion | BT, S | STN | - | - | 15/28 | - | 21 | 18 | 57 | 29 | 25 | 0.935 | 0.852 |
| DT03 | F/68 | 8 | torticollis | I | neck | torsion | BT, AED | GPi | - | - | 16/32 | - | 10 | 18 | 52 | 25 | 16 | 0.769 | 0.815 |
| DT04 | M/45 | 10 | torsion spasm | I | limbs, trunk | torsion | BT, BZ | STN | - | - | 5/18 | - | 13 | 16 | 64 | 29 | 25 | 0.769 | 0.824 |
| DT05 | M/43 | 5 | secondary | CH | limbs | torsion, tremor | BT | STN | - | - | 14/21 | - | 8 | 3 | 81 | 30 | 25 | 0.685 | 0.796 |
| DT06 | F/57 | 8 | torsion spasm | I | trunk | torsion | MR, S, AD | GPi | - | - | 5/12 | - | 18 | 12 | 72 | 30 | 22 | 0.694 | 0.806 |
| DT07 | F/58 | 5 | Meige’s syndrome | I | face, neck, eyelids | spasm | BT, MR, AED | GPi | - | - | 10/27 | - | 10 | 11 | 84 | 27 | 21 | 0.852 | 0.787 |
| DT08 | F/69 | 5 | Meige’s syndrome | I | face, neck, eyelids | spasm | MR, APD | STN | - | - | 8/10 | - | 6 | 5 | 82 | 25 | 16 | 0.528 | 0.519 |
| DT09 | M/56 | 10 | torticollis | I | neck | torsion | BT, MR, S, N, AD | STN | - | - | 8/10 | - | 11 | 20 | 80 | 28 | 30 | 0.908 | 1.000 |
| DT10 | F/59 | 4 | torticollis | I | neck, voice | torsion | BT, MR | STN | - | - | 15/12 | - | 8 | 12 | 65 | 26 | 18 | 0.639 | 0.574 |
| DT11 | M/43 | 10 | blepharospasm | I | eyelids | spasm | BT, MR, BB | STN | - | - | 16/12 | - | 5 | 6 | 56 | 29 | 25 | 0.639 | 0.833 |
| DT12 | F/58 | 1 | torticollis | I | neck | torsion | MR, BZ | GPi | - | - | 13/10 | - | 5 | 3 | 70 | 26 | 18 | 0.870 | 0.889 |
| DT13 | M/45 | 3 | blepharospasm | I | eyelids | spasm | BT, MR, BZ | STN | - | - | 14/29 | - | 8 | 10 | 74 | 29 | 25 | 0.908 | 0.935 |
| DT14 | F/49 | 11 | Meige’s syndrome | I | face, neck, eyelids | spasm | MR, N, S, BZ | GPi | - | - | 9/16 | - | 12 | 9 | 87 | 30 | 25 | 0.769 | 0.861 |
| DT15 | M/57 | 13 | torticollis | I | neck, voice | torsion | MR, AED | GPi | - | - | 8/12 | - | 8 | 12 | 54 | 29 | 24 | 0.815 | 0.787 |
| DT16 | M/48 | 5 | secondary | I | trunk, limbs | torsion | BT, AED, APD | GPi | - | - | 15/12 | - | 5 | 9 | 63 | 24 | 18 | 0.806 | 0.907 |
| DT17 | F/62 | 15 | secondary | CH | limbs | torsion | MR, APD | GPi | - | - | 7/12 | - | 12 | 10 | 63 | 28 | 30 | 0.815 | 0.870 |
| DT18 | F/43 | 5 | torticollis | I | neck, voice | torsion | AD | STN | - | - | 12/15 | - | 24 | 21 | 50 | 27 | 26 | 0.806 | 0.898 |

### *Abbreviations:* AD, antidepressants; AED, antiepileptic agents; APD, anti–Parkinsonian agents; BFMDRS, Burke-Fahn-Marsden dystonia rating scale; BK, bradykinesia; BT, botulinum toxin; BZ, benzodiazepines; CH, cerebral hemorrhage; DT, dystonia; FOG, freezing of gait; HAMA, Hamilton rating scale for anxiety; HAMD, Hamilton rating scale for depression; H-Y scale, Hoehn-Yahr scale; I, idiopathic; LEDD, levodopa equivalent daily dose; MMSE, Mini-Mental State Examination; MoCA, Montreal Cognitive Assessment; MR, muscle relaxants; N, oral narcotics; PD, Parkinson’s disease; S, sedatives; SF-36, short-form health survey 36-item scale; mUPDRS, motor score of the Unified Parkinson’s Disease Rating Scale; mUDRS, motor score of the Unified Dystonia Rating Scale.
